# Supplementary material for: Postural instability and gait disturbance are associated with abnormal stereopsis in Parkinson’s disease
Source: PLoS One. 2025 Jan 23;20(1):e0317935. doi: 10.1371/journal.pone.0317935 (PMC11756755; doi:10.1371/journal.pone.0317935)
Supplement: S1 Table — (DOCX) [file pone.0317935.s001.docx]

**S1 Table.** **Analysis of gait parameters across groups with normal and abnormal stereopsis**

|  | PD_NL  (n = 185) | PD_AS  (n = 55) | *p-*value |
| --- | --- | --- | --- |
| Velocity (cm/s) | 85.41 [82.56 – 88.27] | 83.77 [77.53 – 90.01] | 0.602 |
| Cadence (steps/min) | 106.60 [99.30, 113.60] | 106.10 [100.20, 114.90] | 0.714 |
| Step length differential (cm) | 1.70 [0.81, 3.05] | 2.20 [1.27, 3.22] | 0.124 |
| **Left** |  |  |  |
| Step time (seconds) | 0.56 [0.53, 0.60] | 0.56 [0.51, 0.60] | 0.461 |
| Swing time (seconds) | 0.440 [0.38, 0.42] | 0.39 [0.36, 0.42] | 0.607 |
| Stance time (seconds) | 0.72 [0.68, 0.78] | 0.73 [0.67, 0.79] | 0.864 |
| Single support time (seconds) | 0.40 [0.37, 0.42] | 0.40 [0.37, 0.41] | 0.533 |
| Double support time (seconds) | 0.32 [0.28, 0.37] | 0.33 [0.27, 0.38] | 0.661 |
| Step length (cm) | 48.75 [42.24, 55.10] | 48.20 [38.75, 54.26] | 0.346 |
| Stride length (cm) | 97.86 [85.30, 110.64] | 96.50 [78.36, 106.88] | 0.292 |
| CV of step length | 6.30 [4.90, 8.10] | 6.50 [5.10, 8.10] | 0.600 |
| CV of stride length | 5.00 [4.00, 6.20] | 5.20 [4.30, 6.35] | 0.337 |
| **Right** |  |  |  |
| Step time (seconds) | 0.56 [0.53, 0.60] | 0.56 [0.53, 0.59] | 0.997 |
| Swing time (seconds) | 0.40 [0.37, 0.42] | 0.40 [0.37, 0.42] | 0.817 |
| Stance time (seconds) | 0.73 [0.68, 0.79] | 0.72 [0.66, 0.79] | 0.657 |
| Single support time (seconds) | 0.40 [0.38, 0.42] | 0.39 [0.36, 0.42] | 0.352 |
| Double support time (seconds) | 0.32 [0.29, 0.37] | 0.33 [0.28, 0.38] | 0.683 |
| Step length (cm) | 48.96 [42.82, 55.21] | 47.49 [39.23, 53.27] | 0.251 |
| Stride length (cm) | 98.57 [85.56, 111.32] | 96.46 [78.51, 107.10] | 0.333 |
| CV of step length | 6.20 [4.90, 8.20] | 6.50 [5.05, 8.80] | 0.350 |
| CV of stride length | 5.00 [3.90, 6.40] | 5.00 [4.15, 6.90] | 0.307 |

Values are presented as mean [Confidence interval] or median [Interquatile range]. The P-value was obtained using the Student’s t-test if the data followed a normal distribution; otherwise, the Mann-Whitney U test was used. Abbreviations: PD_NL = Parkinson’s disease with normal stereopsis; PD_AS = Parkinson’s disease with abnormal stereopsis; CV = Coefficient of Variation.
