# Supplementary material for: Postural instability and gait disturbance are associated with abnormal stereopsis in Parkinson’s disease
Source: PLoS One. 2025 Jan 23;20(1):e0317935. doi: 10.1371/journal.pone.0317935 (PMC11756755; doi:10.1371/journal.pone.0317935)
Supplement: Attachment — Submitted filename: Response to Reviewers.docx [file pone.0317935.s002.docx]

Emily Chenette, Ph.D.

Editor-In-Chief

*PLOS One*

October 1st, 2024

Dear Chenette:

On behalf of the authors, I would like to thank you and the reviewers for the valuable and helpful comments on our submitted manuscript, “**Postural instability and gait disturbance are associated with abnormal stereopsis in Parkinson's disease: A retrospective study of 240 patients**” [PONE-D-24-29202].

We have carefully reviewed the comments and have revised the manuscript accordingly. Our responses are provided in a point-by-point manner, and changes to the manuscript are shown in yellow highlight, which are also visible through the Track Changes function.

We would be happy to make further corrections if necessary and look forward to hearing from you soon.

Sincerely,

Seong-Beom Koh, MD, PhD.

Professor, Department of Neurology and Parkinson’s Disease Center

Korea University Guro Hospital

Korea University College of Medicine

148, Gurodong-ro, Guro-gu, Seoul 08308, Republic of Korea

Tel: +82-2-2626-3169, Fax: +82-2-2626-1257, Email: parkinson@korea.ac.kr

Response to Reviewer 1

Comments to the Author

This study is an interesting study with a well-designed structure. Still visual symptoms in PD are under-estimated. Especially, the authors recruited quite big number of drug-naive PD patients in this study, so they could exclude possible confounders, mainly seen in the advanced stage.

Thank you for your review of our paper. We have answered each of your points below.

**Comment 1:** As mentioned before, enrollment of drug-naive PD patients can be a big strength of this study. However, the authors did not describe why they recruited drug-naive PD patients in the introduction section, so I recommend emphasizing the reason why they included drug-naive PD patients in introduction section and maybe in discussion section. ­

**Response 1:** We greatly appreciate the insightful comment provided by the reviewer. We first apologize for the typo "*de novo*" in the abstract. This study enrolled patients who were newly diagnosed with Parkinson's disease at our hospital, and it remains uncertain whether these patients had taken anti-parkinsonian medications. The patients included in this study consist of a combination of drug-naive individuals and those who started medication within the past month. Therefore, we thought it necessary to revise “*de novo*” to “early.” According to the prior study (Sun et al., 2014), there was no significant difference in stereopsis between the on and off states. Nevertheless, to minimize the potential influence of medication, if the patients were on medication, we discontinued their medications during the initial work-up, including stereopsis tests and UPDRS assessments, and conducted the tests in the off state.

**Abstract**

Methods: We designed a cross-sectional study and included 240 early PD patients without ophthalmologic problems other than abnormal stereopsis.

**Materials and Methods**

A total of 240 patients newly diagnosed with PD during this period were included in this study.

Data were collected through reviewing electronic medical records between March 1, 2021 and March 31, 2021. The personal information was kept confidential, and each individual was assigned anonymously. All data were entered into the electronic medical record at the time of the patients' initial diagnosis of PD. Two neurologists collected the data from medical records including clinical information such as histories, disease duration, symptoms, neurological examination results, scale scores, neurophysiological test results, cognitive function test (components of the Seoul Neuropsychological Screening Battery, and the Mini Mental State Examination) results and stereopsis test results. All tests and scores were conducted in the off state within one month of the patient’s diagnosis.

**Comment 2:** Some of the enrolled subjects may be diagnosed as atypical parkinsonism later. How long did the authors follow up after the diagnosis of PD? In particular, freezing of gait (FOG) is very rare in drug-naive PD patients, but can be seen in patients with atypical parkinsonism even from the early stage. In this study 7% of enrolled subjects had FOG, and it is better to check the data again.

**Response 2:** We agree that the symptom of freezing of gait (FOG) is very rare in early PD. The FOG we assessed was recorded as a binary item based on the patient’s self-report, which we believe may have lower reliability. We reviewed the medical records again in accordance with the reviewer's comments. But the follow-up period varied for each patient, and in some cases, it was difficult to track patients due to follow-up loss. It was not possible to definitively confirm whether the patient's final diagnosis had changed. Therefore, we decided to remove the FOG variable due to its low reliability.

**Comment 3:** The authors checked various symptoms, including constipation, anosmia, orthostatic dizziness, urinary incontinence, visual hallucination, easy falling and FOG. How did they define these symptoms? Any scale used to screen these symptoms (e.g. NMSS sub-items)?

**Response 3:** We thank the reviewer for the comment. Constipation, anosmia, orthostatic dizziness, urinary incontinence, visual hallucinations, easy falling, and FOG were recorded based on a simple yes or no format rather than using a validated questionnaire. Therefore, we replaced “constipation” with NMSS domain 6 (gastrointestinal tract), “orthostatic dizziness” with NMSS domain 1 (cardiovascular including falls), “urinary incontinence” with NMSS domain 7 (urinary), and “visual hallucinations” with NMSS domain 4 (perceptual problems/hallucinations) subscales. “Anosmia”, “FOG”, and “easy falling” were removed from the analysis.

**Table 1.** Demographic and clinical characteristics of enrolled patients

|  | PD_NL  (n = 185) | PD_AS  (n = 55) | Total  (n = 240) | *p-* value |
| --- | --- | --- | --- | --- |
| Demographics |  |  |  |  |
| Age, y | 69.7 [68.57 – 70.83] | 70.0 [68.01 – 72.06] | 69.8 [68.80 – 70.76] | 0.779 |
| Sex, female | 99 (53.5%) | 27 (49.1%) | 126 (52.5%) | 0.672 |
| Education, y | 9.0 [6.0; 12.0] | 9.0 [6.0; 12.0] | 9.0 [6.0; 12.0] | 0.787 |
| Hypertension | 83 (44.9%) | 21 (38.2%) | 104 (43.3%) | 0.470 |
| Diabetes | 36 (19.5%) | 11 (20.0%) | 47 (19.6%) | 1.000 |
| Clinical characteristics |  |  |  |  |
| Disease duration, m | 12.0 [7.0;30.0] | 18.0 [8.0; 36.0] | 12.0 [7.0; 36.0] | 0.347 |
| **H&Y stage** | **2.0 [2.0;** **2.0]** | **2.0 [2.0; 2.5]** | **2.0 [2.0; 2.5]** | **0.024** |
| UPDRS part Ⅰ | 2.0 [1.0; 3.0] | 2.0 [1.0; 3.0] | 2.0 [1.0; 3.0] | 0.547 |
| UPDRS part Ⅱ | 8.0 [5.0; 9.0] | 8.0 [6.0; 11.5] | 8.0 [5.0; 10.0] | 0.227 |
| UPDRS part Ⅲ | 23.0 [17.0; 29.0] | 24.0 [18.0; 28.0] | 23.0 [17.5; 29.0] | 0.529 |
| Subtype  Tremor dominant  PIGD  Intermediate | 56 (30.3%)  101 (54.6%)  28 (15.1%) | 17 (30.9%)  29 (52.7%)  9 (16.4%) | 73 (30.4%)  130 (54.2%)  37 (15.4%) | 0.963 |
| NMSS domain 1: Cardiovascular including falls | 0.0 [0.0; 1.0] | 0.0 [0.0; 1.0] | 0.0 [0.0; 1.0] | 0.226 |
| NMSS domain 4: Perceptual problems/hallucinations | 0.0 [0.0; 0.0] | 0.0 [0.0; 0.0] | 0.0 [0.0; 0.0] | 0.592 |
| NMSS domain 6: Gastrointestinal tract | 1.0 [0.0; 3.0] | 1.0 [0.0; 3.5] | 1.0 [0.0; 3.0] | 0.786 |
| NMSS domain 7: urinary | 5.0 [1.0; 7.0] | 5.0 [2.5; 9.0] | 5.0 [1.0; 8.0] | 0.283 |
| NMSS total scoore | 27.0 [18.0; 38.0] | 27.0 [13.5; 39.0] | 27.0 [17.0; 38.0] | 0.806 |
| Tilt table test (Orthostatic hypotension %) | 57 (30.8%) | 20 (36.4%) | 77 (32.1%) | 0.542 |
| MMSE (abnormal %) | 39 (21.1%) | 15 (27.3%) | 54 (22.5%) | 0.434 |
| Digit Span Test backward (z-score) | -0.2 [-0.8; 0.4] | -0.3 [-0.9; 0.6] | -0.2 [-0.9; 0.4] | 0.982 |
| **RCFT copy (z-score)** | **0.3 [-0.6; 0.8]** | **-0.3 [-1.1; 0.6]** | **0.2 [-0.7; 0.8]** | **0.032** |
| SVLT delayed recall (z-score) | -0.6 [-0.75 – -0.43] | -0.7 [-0.99 – -0.37] | -0.6 [-0.75 – -0.47] | 0.589 |
| RCFT delayed recall (z-score) | -0.5 [-1.2; 0.4] | -0.6 [-1.1; 0.3] | -0.5 [-1.1; 0.4] | 0.685 |
| COWAT animal (z-score) | -0.3 [-0.41 – -0.1] | -0.3 [-0.59 – -0.07] | -0.3 [-0.41 – -0.14] | 0.645 |
| COWAT phonemic (z-score) | -0.4 [-1.0; 0.3] | -0.5 [-1.2; 0.1] | -0.4 [-1.0; 0.2] | 0.249 |
| Stroop Color Reading (z-score) | -0.2 [-1.1; 0.7] | -0.6 [-1.7; 0.5] | -0.3 [-1.3; 0.7] | 0.101 |

**Comment 4:** In terms of table 2, how about using sub-score of UPDRS part 3 (e.g. sub-score for tremor, rigidity, bradykinesia and axial symptoms)? This would be more easy for the readers.

**Response 4:** We thank the reviewer for the thoughtful comment. We have revised the table in accordance with the reviewer's suggestions.

**Table 2.** The ~~item~~ subscales of UPDRS part III in enrolled patients

|  | PD_NL  (n = 185) | PD_AS  (n = 55) | *p-* value |
| --- | --- | --- | --- |
| Subscale of axial and gait | 5.00 [4.00, 6.00] | 6.00 [4.00, 8.00] | 0.063 |
| Subscale of resting tremor | 1.00 [0.00, 3.00] | 1.00 [0.00, 3.00] | 0.892 |
| Subscale of postural tremor | 1.00 [0.00, 2.00] | 1.00 [0.00, 2.00] | 0.311 |
| Subscale of tremor | 2.00 [1.00, 4.00] | 2.00 [0.50, 5.00] | 0.601 |
| Subscale of rigidity | 4.00 [2.00, 6.00] | 4.00 [2.00, 6.00] | 0.702 |
| Subscale of bradykinesia left | 6.00 [4.00, 8.00] | 6.00 [4.50, 7.00] | 0.804 |
| Subscale of bradykinesia right | 5.00 [3.00, 7.00] | 5.00 [4.00, 7.00] | 0.350 |
| Subscale of bradykinesia both | 10.81 [10.17 – 11.44] | 11.18 [9.92 – 12.44] | 0.380 |
| **UPDRS III sum** | 23.00 [17.00, 29.00] | 24.00 [18.00, 28.00] | 0.528 |

**Comment 5:** Results without statistical significance like the comparison of GAITRITE data can be moved to supplement.

**Response 5:** We agree with the reviewer’s suggestion to move Table 3 to Supplement table 1.

**Supporting information**

**S1 Table.** **Analysis of gait parameters across groups with normal and abnormal stereopsis**

|  | PD_NL  (n = 185) | PD_AS  (n = 55) | *p-*value |
| --- | --- | --- | --- |
| Velocity (cm/s) | 85.41 [82.56 – 88.27] | 83.77 [77.53 – 90.01] | 0.602 |
| Cadence (steps/min) | 106.60 [99.30, 113.60] | 106.10 [100.20, 114.90] | 0.714 |
| Step length differential (cm) | 1.70 [0.81, 3.05] | 2.20 [1.27, 3.22] | 0.124 |
| **Left** |  |  |  |
| Step time (seconds) | 0.56 [0.53, 0.60] | 0.56 [0.51, 0.60] | 0.461 |
| Swing time (seconds) | 0.440 [0.38, 0.42] | 0.39 [0.36, 0.42] | 0.607 |
| Stance time (seconds) | 0.72 [0.68, 0.78] | 0.73 [0.67, 0.79] | 0.864 |
| Single support time (seconds) | 0.40 [0.37, 0.42] | 0.40 [0.37, 0.41] | 0.533 |
| Double support time (seconds) | 0.32 [0.28, 0.37] | 0.33 [0.27, 0.38] | 0.661 |
| Step length (cm) | 48.75 [42.24, 55.10] | 48.20 [38.75, 54.26] | 0.346 |
| Stride length (cm) | 97.86 [85.30, 110.64] | 96.50 [78.36, 106.88] | 0.292 |
| CV of step length | 6.30 [4.90, 8.10] | 6.50 [5.10, 8.10] | 0.600 |
| CV of stride length | 5.00 [4.00, 6.20] | 5.20 [4.30, 6.35] | 0.337 |
| **Right** |  |  |  |
| Step time (seconds) | 0.56 [0.53, 0.60] | 0.56 [0.53, 0.59] | 0.997 |
| Swing time (seconds) | 0.40 [0.37, 0.42] | 0.40 [0.37, 0.42] | 0.817 |
| Stence time (seconds) | 0.73 [0.68, 0.79] | 0.72 [0.66, 0.79] | 0.657 |
| Single support time (seconds) | 0.40 [0.38, 0.42] | 0.39 [0.36, 0.42] | 0.352 |
| Double support time (seconds) | 0.32 [0.29, 0.37] | 0.33 [0.28, 0.38] | 0.683 |
| Step length (cm) | 48.96 [42.82, 55.21] | 47.49 [39.23, 53.27] | 0.251 |
| Stride length (cm) | 98.57 [85.56, 111.32] | 96.46 [78.51, 107.10] | 0.333 |
| CV of step length | 6.20 [4.90, 8.20] | 6.50 [5.05, 8.80] | 0.350 |
| CV of stride length | 5.00 [3.90, 6.40] | 5.00 [4.15, 6.90] | 0.307 |

**Comment 6:** Did the authors check RBD or dysautonomia (not just symptoms but autonomic function test or orthostatic hypotension)? Considering these symptoms can be markers to differentiate brain-first vs. body-first subtype, analysis with these variables can be also interesting.

**Response 6:** We thank the reviewer for the comment. Since we do not perform polysomnography as a routine test, we only have symptom history for RBD. However, we have results from the tilt table test, which inserted into the table 1.

**Table 1.** Demographic and clinical characteristics of enrolled patients

|  | PD_NL  (n = 185) | PD_AS  (n = 55) | Total  (n = 240) | *p-* value |
| --- | --- | --- | --- | --- |
| Demographics |  |  |  |  |
| Age, y | 69.7 [68.57 – 70.83] | 70.0 [68.01 – 72.06] | 69.8 [68.80 – 70.76] | 0.779 |
| Sex, female | 99 (53.5%) | 27 (49.1%) | 126 (52.5%) | 0.672 |
| Education, y | 9.0 [ 6.0;12.0] | 9.0 [ 6.0;12.0] | 9.0 [6.0;12.0] | 0.787 |
| Hypertension | 83 (44.9%) | 21 (38.2%) | 104 (43.3%) | 0.470 |
| Diabetes | 36 (19.5%) | 11 (20.0%) | 47 (19.6%) | 1.000 |
| Clinical characteristics |  |  |  |  |
| Disease duration, m | 12.0 [ 7.0;30.0] | 18.0 [ 8.0;36.0] | 12.0 [7.0;36.0] | 0.347 |
| **H&Y stage** | **2.0 [2.0;** **2.0]** | **2.0 [2.0; 2.5]** | **2.0 [2.0; 2.5]** | **0.024** |
| UPDRS part Ⅰ | 2.0 [ 1.0; 3.0] | 2.0 [ 1.0; 3.0] | 2.0 [ 1.0; 3.0] | 0.547 |
| UPDRS part Ⅱ | 8.0 [ 5.0; 9.0] | 8.0 [ 6.0;11.5] | 8.0 [ 5.0;10.0] | 0.227 |
| UPDRS part Ⅲ | 23.0 [17.0;29.0] | 24.0 [18.0;28.0] | 23.0 [17.5;29.0] | 0.529 |
| Subtype  Tremor dominant  PIGD  Intermediate | 56 (30.3%)  101 (54.6%)  28 (15.1%) | 17 (30.9%)  29 (52.7%)  9 (16.4%) | 73 (30.4%)  130 (54.2%)  37 (15.4%) | 0.963 |
| NMSS domain 1: Cardiovascular including falls | 0.0 [0.0;1.0] | 0.0 [0.0; 1.0] | 0.0 [0.0; 1.0] | 0.226 |
| NMSS domain 4: Perceptual problems/hallucinations | 0.0 [0.0; 0.0] | 0.0 [0.0; 0.0] | 0.0 [0.0; 0.0] | 0.592 |
| NMSS domain 6: Gastrointestinal tract | 1.0 [0.0; 3.0] | 1.0 [0.0; 3.5] | 1.0 [0.0; 3.0] | 0.786 |
| NMSS domain 7: urinary | 5.0 [1.0; 7.0] | 5.0 [2.5; 9.0] | 5.0 [1.0; 8.0] | 0.283 |
| NMSS total score | 27.0 [18.0; 38.0] | 27.0 [13.5; 39.0] | 27.0 [17.0; 38.0] | 0.806 |
| Tilt table test (Orthostatic hypotension %) | 57 (30.8%) | 20 (36.4%) | 77 (32.1%) | 0.542 |
| MMSE (abnormal %) | 39 (21.1%) | 15 (27.3%) | 54 (22.5%) | 0.434 |
| Digit Span Test backward (z-score) | -0.2 [-0.8; 0.4] | -0.3 [-0.9; 0.6] | -0.2 [-0.9; 0.4] | 0.982 |
| **RCFT copy (z-score)** | **0.3 [-0.6; 0.8]** | **-0.3 [-1.1; 0.6]** | **0.2 [-0.7; 0.8]** | **0.032** |
| SVLT delayed recall (z-score) | -0.6 [-0.75 – -0.43] | -0.7 [-0.99 – -0.37] | -0.6 [-0.75 – -0.47] | 0.589 |
| RCFT delayed recall (z-score) | -0.5 [-1.2; 0.4] | -0.6 [-1.1; 0.3] | -0.5 [-1.1; 0.4] | 0.685 |
| COWAT animal (z-score) | -0.3 [-0.41 – -0.1] | -0.3 [-0.59 – -0.07] | -0.3 [-0.41 – -0.14] | 0.645 |
| COWAT phonemic (z-score) | -0.4 [-1.0; 0.3] | -0.5 [-1.2; 0.1] | -0.4 [-1.0; 0.2] | 0.249 |
| Stroop Color Reading (z-score) | -0.2 [-1.1; 0.7] | -0.6 [-1.7; 0.5] | -0.3 [-1.3; 0.7] | 0.101 |

Thank you for your kind and helpful comments.

Response to Reviewer 2

Comments to the Author

This study is original and relevant in exploring the relationship between postural instability, gait disturbances, and stereopsis abnormalities in patients with Parkinson's disease. This topic has been little investigated to date. The results presented are interesting, suggesting a possible association between the PIGD subtype and visual dysfunction, which may open new perspectives on the impact of visual abnormalities on the disease's motor symptoms. However, despite the significant contribution, some aspects could be improved to strengthen the conclusions and increase the robustness of the findings.

Thank you for reviewing our manuscript. Our answers to your queries are as follows:

**Comment 1:** The most critical aspect is the absence of significant differences in the objective gait parameters between the groups. Can the relationship between gait/postural alterations and stereopsis be sustained based on scores in some items of UPDRS-section III?

**Response 1:** We thank the reviewer for the thoughtful comment. We agree that the lack of significant differences in objective gait parameters represents a major limitation of this study. As mentioned in the discussion, the GaitRite system has limitations in measuring certain kinetics and kinematics parameters such as upper extremity motion and center of pressure. It is possible that subtle differences not captured by this equipment may be reflected in the UPDRS scores, which are based on human assessment. Therefore, considering the value of examinations conducted by trained experts, we consider that conclusions based on UPDRS-section III items remain valid.

**Discussion**

Second, additional gait analysis using GaitRite did not reveal any differences between the two groups. GaitRite, which we used to analyze gait quantitatively, can assess spatiotemporal parameters; but GaitRite is limited in its ability to assess ~~postural instability and upper limb movements~~ certain kinetics and kinematics parameters such as upper extremity motion and center of pressure. It is possible that subtle differences not captured by this equipment may be reflected in the UPDRS scores, which are based on human assessment.

**Comment 2: (**Introduction) The introduction situates the study's relevance in the context of PD, mentioning stereopsis as an important non-motor symptom. However, the article could delve deeper into how visual dysfunction, specifically stereopsis, contributes to motor disturbances in PD. This more comprehensive exploration is crucial for providing a solid justification for the study and understanding the full impact of visual abnormalities on the disease's motor symptoms.

**Response 2:** We appreciate the insightful comment provided by the reviewer. First, we would like to apologize for any confusion caused by ambiguous expressions in the introduction. Our study results indicated that patients with higher PIGD scores had a higher risk of abnormal stereopsis. Since stereopsis is one of the sensory input information, it is believed that this information could also affect gait disturbance and posture, potentially creating a vicious cycle. Additionally, both the PIGD score and abnormal stereopsis may be associated with cholinergic nerve degeneration. We clarified this point and revised the introduction.

**Introduction**

Also, patients with abnormal stereopsis have worse bradykinesia compared to those with normal stereopsis [7]. It has been observed that the group of PD with freezing of gait demonstrated worse stereoacuity compared to the group without this symptom [8]. However, researchers have not yet to ~~uncover~~ elucidated the specific pathways as to why abnormal stereopsis occurs in PD patients, or ~~through which these~~ how it is related to ~~stereopsis abnormalities exacerbate~~ motor symptoms.

The pedunculopontine nucleus (PPN) plays a crucial role in gait disturbance and posture in PD. It is also an area that affects freezing of gait, a disabling symptom uniquely found in PD [9]. This region consists of neurons projecting both ascending and descending between the cortex, thalamus, basal ganglia, cerebellum, and spinal cord, performing various functions [10]. Among these, PPN cholinergic projections have an extended effect on midbrain dopaminergic systems by innervating neurons in the substantia nigra pars compacta and ventral tegmental area [10]. By modulating dopamine release, it can also influence striatal input from the cortex and thalamus [10].

The neurotransmitter balance in peripheral organs, such as the retina, also affects stereopsis. The cholinergic system and dopaminergic deficits in the retina are involved in interocular interaction and mixed percepts by regulating the balance of excitatory and inhibitory signals in the binocular visual cortex [11]. Abnormal stereopsis can be affected by decreased visual acuity of one eye and decreased color perception [3, 7]. ~~Dopamine deficiency in peripheral organs, such as the retina, is associated with abnormalities in stereopsis [7].~~ A dopamine deficit in the retina and other structures of the peripheral visual system can impair visual function, including stereopsis [12, 13].

**Comment 3:** (Methods) The adopted test to detect stereopsis is appropriate, but the authors do not mention its sensitivity and specificity in patients with PD. Please consider including this information to improve the interpretation of the results.

**Response 3:** We thank the reviewer for the excellent comment. We do not have the raw data from the stereopsis tests conducted on the control group, so it would be difficult to present sensitivity and specificity. However, we will reference previous studies conducted in the same lab [reference 6 and 14] and other relevant research [reference 17] in the manuscript.

**Materials & Methods**

Normal stereoscopic vision stereopsis was characterized as having an arc measurement of 60 seconds or less in the Titmus fly test [6, 14, 17].

**Comment 4:** (Results and Discussion) The results show differences between the groups regarding postural instability and gait according to UPDRS section III. However, the objective gait analysis did not show significant differences between the groups, even in parameters closely related to postural and gait alterations such as single and double support time and CV of step and stride length. Are the scores in UPDRS items used to classify the PIGD subtype correlated with these gait parameters? In the absence of differences in objective gait measures, further explanation is needed for the association between PIGD and abnormal stereopsis.

**Response 4:** We thank the reviewer for the excellent comment. We have added a table showing the correlation between the parameters measured in the gait analysis and the PIGD score (Table 3).

**Table 3.** The correlations between the PIGD score and parameters of gait analysis

|  | Correlations with PIGD score | |  |
| --- | --- | --- | --- |
|  | Correlation coefficient (rho) | *P*-value | |
| Velocity (cm/s) | **-0.455** | **<0.001** | |
| Cadence (steps/min) | -0.108 | 0.094 | |
| Step length differential (cm) | 0.117 | 0.071 | |
| Left step time (seconds) | 0.103 | 0.112 | |
| Right step time (seconds) | 0.071 | 0.277 | |
| Left swing time (seconds) | -0.092 | 0.154 | |
| Right swing time (seconds) | **-0.139** | **0.032** | |
| Left stance time (seconds) | **0.200** | **0.002** | |
| Right stance time (seconds) | **0.221** | **0.001** | |
| Left single support time (seconds) | **-0.162** | **0.012** | |
| Right single support time (seconds) | -0.116 | 0.074 | |
| Left double support time (seconds) | **0.339** | **<0.001** | |
| Right double support time (seconds) | **0.321** | **<0.001** | |
| Left step length (cm) | **-0.480** | **<0.001** | |
| Right step length (cm) | **-0.470** | **<0.001** | |
| Left stride length (cm) | **-0.485** | **<0.001** | |
| Right stride length (cm) | **-0.482** | **<0.001** | |
| Left CV of step length | **0.354** | **<0.001** | |
| Right CV of step length | **0.411** | **<0.001** | |
| Left CV of stride length | **0.374** | **<0.001** | |
| Right CV of stride length | **0.409** | **<0.001** | |

**Comment 5:** (Results and Discussion) The study found a significant link between the PIGD subtype and abnormal stereopsis. However, based on postural control theories, the discussion regarding the connection between the PIGD subtype and stereopsis abnormalities must be deeper. The authors also suggested that cholinergic denervation in PD may be associated with abnormal stereopsis, but the explanation is unclear and does not effectively connect the study results to this theory.

**Response 5:** We agree with the reviewer’s comment. We tried a more detailed discussion regarding cholinergic denervation. The impairment of posture that may occur due to abnormal multisensory input caused by abnormal stereopsis could induce a vicious cycle, potentially further exacerbating posture and gait.

**Discussion**

Physiologically, cholinergic denervation is a major cause of PIGD features in PD [20]. The PPN, which plays a key role in posture and gait stability ~~[21]~~, is part of the PPN-laterodorsal tegmental complex and sends cholinergic projections to the thalamus [20]. This area is known to play an active role in sensorimotor control [21, 22]. There is a possibility that these projections are associated with abnormal stereopsis. Also, p~~P~~revious study has~~ve also~~ indicated that the cholinergic system is involved in stereopsis abnormalities [11]. The cholinergic system regulates the excitatory and inhibitory balance in the binocular visual cortex, and cholinergic enhancement in the visual cortex (V1) decreases inhibitory drive. This increases interocular interaction and mixed percepts [11]. Therefore, cholinergic denervation in PD may not only increase the risk of postural instability and gait disturbance but also increase the risk of stereopsis abnormalities. Additionally~~, integrating various sensory inputs is crucial for the motor planning of posture and gait, and stereopsis is one such sensory input [23].~~ abnormal stereopsis may lead to a qualitative degradation of visual information, potentially interfering with feedback and adaptation processes. This can further create a vicious cycle by negatively impacting posture and gait.

**Comment 6: (**Study limitations) Please make sure to include the unique cohort as one of the limitations to consider.

**Response 6:** Thanks for the reviewer’s comment. In response to the reviewer's comments, we have revised the discussion.

**Discussion**

First, its retrospective study design and its being conducted at a single tertiary hospital are limitations. Additionally, it is possible that the enrolled patients constituted a unique cohort. Therefore, the generalizability of our results may be limited.

Thank you for your kind and helpful comments.

Response to Reviewer 3

Comments to the Author

Thank you for reviewing of our manuscript. Our answers to your queries are as follows:

**Comment 1:** The title is informative; however, it is long. The authors should consider shortening the title, and avoiding using the term retrospective because it is no longer recommended. Besides, this is a cross-sectional study, no matter if it uses data from a databank or if the authors seek the data prospectively.

**Response 1:** We agree with the reviewer’s thoughtful comment. We changed the title to “Postural instability and gait disturbance are associated with abnormal stereopsis in Parkinson's disease.”

**Title**

Postural instability and gait disturbance are associated with abnormal stereopsis in Parkinson's disease

**Comment 2: (**Abstract) The text should be shortened without losing information. The authors should insert the setting and the study design.

**Response 2:** Thank you for providing the insight. We added “designed a cross-sectional study and” in the Methods section of abstract.

**Abstract - Methods**

We designed a cross-sectional study and included 240 early PD patients without ophthalmologic problems other than abnormal stereopsis.

**Comment 3:** **(**Abstract) The main and secondary outcomes should be clearly stated.

**Response 3:** Thanks for the reviewer’s comment. We revised the background section of the abstract.

**Abstract - Background**

Visual dysfunction, including abnormal stereopsis, is a significant non-motor symptom in Parkinson’s disease (PD) that can reduce quality of life and appears early in the disease. Abnormal stereopsis is associated with worse bradykinesia and freezing of gait, though the exact pathways linking stereopsis to motor symptoms remain unclear. In this study, we hypothesized that a high postural instability and gait disturbance score would increase the risk of abnormal stereopsis in PD. Furthermore, in PD patients, the pedunculopontine nucleus and laterodorsal tegmental complex play an active role in sensorimotor control, and these areas provide cholinergic projections. Cholinergic degeneration may be associated with symptoms such as abnormal stereopsis, postural instability, gait disturbances and cognitive impairment. Therefore, in this study, we hypothesized that a high postural instability and gait disturbance score would increase the risk of abnormal stereopsis in PD. ~~There are various non-motor symptoms in Parkinson's disease (PD), and ocular symptoms are among these. Ocular symptoms, including abnormalities in stereopsis, the perception of depth created by the brain’s processing of binocular vision, are highlighted as prodromal symptoms. Previous studies have reported higher Unified Parkinson’s Disease Rating Scale (UPDRS) motor scores in patients with abnormal stereopsis. In this study, we analyzed the differences in symptoms and test results between PD patients with and without stereopsis abnormalities. We also identified factors that increase the risk of abnormal stereopsis in PD.~~

**Comment 4:** **(**Abstract) It is unclear what data was retrieved from the patient record. Were the patients examined routinely for the stereopsis diagnosis? In which moment? This must be clarified.

**Response 4:** We agree with the reviewer’s thoughtful comment. We performed the stereopsis test only once at the time of the patient's initial Parkinson's disease diagnosis. We added “Stereopsis testing was performed only once at the time of the patient’s initial PD diagnosis.” In the method section of abstract.

**Abstract - Methods**

To evaluate stereopsis, we used Titmus stereo test plates. Stereopsis testing was performed only once at the time of the patient’s initial PD diagnosis.

**Comment 5:** **(**Abstract) The results lack measures of association from the logistic regression – please state also the confidence interval.

**Response 5:** We agree with the reviewer’s thoughtful comment. We added “(95% confidence interval: 1.37-5.15)” in the Result section of abstract.

**Abstract -Results**

Among the total of 240 PD patients, 185 were in the normal stereopsis group and 55 were in the abnormal stereopsis group. ~~When comparing each component of UPDRS part 3, the two groups differed in the items ‘arising from chair,’ ‘postural instability,’ and ‘gait’.~~ In the ~~stepwise logistic regression~~ analysis for risk factors related to abnormal stereopsis, the postural instability and gait difficulties (PIGD) subtype score was significantly associated with abnormal stereopsis. (95% confidence interval: 1.37-5.15)

**Comment 6:** (Introduction) The text offers the reader a sufficient amount of information about stereopsis. Nevertheless, the flow of ideas is confusing across the paragraphs. Please consider combining the first two paragraphs to provide essential information on the prevalence of stereopsis in PD and its associations with other symptoms already defined in the literature.

**Response 6:** We have incorporated the reviewer’s comment by combining the first two paragraphs.

**Introduction**

Visual dysfunction is among the various non-motor symptoms in Parkinson’s disease (PD) [1-5]. Visual dysfunction in PD, which can be identified in the prodromal stage, can significantly reduce quality of life [4, 5]. One specific aspect of visual dysfunction in PD is abnormal stereopsis, a problem with the perception of depth created by the brain’s processing of binocular vision.

**Comment 7:** (Introduction) The second paragraph could be better organized when considering the pathophysiology of stereopsis in PD. Suggestion: begin with the neurochemical dysfunctions associated with stereopsis and finish with the findings in functional MRI.

**Response 7:** In response to the reviewer’s suggestions, we have revised the introduction.

**Comment 8:** (Introduction) Finally, state the study's specific objectives, including any prespecified hypotheses based on the motivations for this research.

**Response 8:** We thank the reviewer for the comment. In response to the reviewer’s suggestion, we have clariﬁed the study’s objective in the final paragraph of the introduction.

**Introduction**

This study was performed to analyze the differences in symptoms and various test results between PD patients with abnormal stereopsis and those without and to identify the factors that increase the risk of abnormal stereopsis. Specifically, to explore the relationship between posture, gait, and stereopsis, all of which involve PPN cholinergic projections, we hypothesized that a high postural instability and gait disturbance score would increase the risk of abnormal stereopsis in PD.

**Comment 9:** (Methods) Line 109: remove the term “retrospective”.

**Response 9:** We have removed the term “retrospective” to reflect the reviewer’s comment.

**Materials and methods**

This was a ~~retrospective~~ cross-sectional study conducted at the Korea University Guro Hospital, a tertiary center in Seoul, Korea, from January 1, 2016 to December 31, 2020.

**Comment 10:** (Methods) Line 116: Patients with secondary parkinsonism (vascular parkinsonism, which presents as a severe white matter hyperintensity burden in magnetic resonance imaging and hydrocephalus): the information regarding MRI findings in vascular parkinsonism is irrelevant and excessive in the context of the study. The authors should consider removing it.

**Response 10:** We agree with the reviewer’s comment. We have removed “, which presents as a severe white matter hyperintensity burden in magnetic resonance imaging” to reflect the reviewer’s comment.

**Materials and methods**

Patients with secondary parkinsonism (vascular parkinsonism ~~which presents as a severe white matter hyperintensity burden in magnetic resonance imaging~~ and hydrocephalus) and tumor also excluded.

**Comment 11:** (Methods) Line 141: there is a typographic error in the phrase, please review

**Response 11:** We revised “stereoscopic vision” to “stereopsis” to reflect the reviewer’s comment.

**Materials and methods**

Normal ~~stereoscopic vision~~ stereopsis was characterized as having an arc measurement of 60 seconds or less in the Titmus fly test [6, 14, 17].

**Comment 12:** (Methods) The patients underwent visual and gait analysis. Was this done at the same time, or at least with a small interval? What was the interval between the analyses, considering the impact of stereopsis on gait performance? This is crucial information regarding potential measurement biases.

**Response 12:** We agree with the reviewer’s thoughtful comment. All patients underwent visual and gait analysis at the same day with off state.

**Materials and methods**

Stereopsis was assessed ~~by~~ using Titmus stereo test plates on the same day as the gait analysis and UPDRS assessment (Stereo Optical Co. Inc., Chicago, Illinois, USA).

**Comment 13:** (Methods) Line 153: the term "statistically significant" in the text (p-values < 0.05 were considered significant.) is no longer recommended since all p-values come from a statistical analysis, which is redundant. Please remove the term "statistically."

**Response 13:** We have removed the term “statistically” to reflect the reviewer’s comment.

**Materials and methods**

*p*-values < 0.05 were considered ~~statistically~~ significant.

**Comment 14:** (Methods) The study design has been defined; however, it lacks statements about primary and secondary outcomes, covariates considered as possible confounders, and how the authors addressed missing data

**Response 14:** Thank you for providing these insights. We have added this information to the revised version.

**Materials and methods**

Outcomes, covariates, missing data

Our primary outcome was the presence of abnormal stereopsis in patients with PD. The secondary outcome was subjective autonomic symptoms, and the covariates included age, sex, hypertension, diabetes, duration of symptoms, presence of orthostatic hypotension, and results of cognitive function tests related to vision. Because only patients who completed all tests were included, there was no missing data.

**Comment 15:** (Results) Line 163: The Hoehn & Yahr scale is ordinal and should not be summarized using means and SD, but rather medians and interquartile. Additionally, the statistical test should be suitable for ordinal data.

**Response 15:** We agree that following the reviewer's suggestion is in line with the appropriate principles. Initially, we presented the data as mean and standard deviation, as we believed that using the median and interquartile range did not adequately capture the characteristics of the two groups. (Initially, p-values were calculated using the Mann-Whitney U test, not Student’s t-test.) In response to the reviewer’s comments, we have revised the data presentation from mean and standard deviation to median and interquartile range.

**Table 1.**

| **H&Y stage** | **2.0 [2.0;** **2.0]** | **2.0 [2.0; 2.5]** | **2.0 [2.0; 2.5]** | **0.024** |
| --- | --- | --- | --- | --- |

**Comment 16:** (Results) Table 1 contains not only demographic data but also a statistical analysis comparing the groups, so the table titles should be adjusted.

**Response 16:** We have rewritten the table title of Table 1 to be more in line with your comments.

**Table 1.** Demographic~~es~~ and clinical characteristics of enrolled~~study~~ patients

**Comment 17:** (Results) Table 1 also lacks the statistical tests used in each analysis.

**Response 17:** We sincerely apologize for the inadvertent omission of this information. In accordance with the recommendation, we have incorporated the following statement: "For the comparison of baseline characteristics, the Shapiro-Wilk normality test was conducted for continuous variables to assess normality. If the data followed a normal distribution, the Student's t-test was applied; otherwise, the Mann-Whitney U test was used. For categorical variables, the Chi-square test or Fisher's exact test was used."

**Materials and methods**

For the comparison of baseline characteristics, the Shapiro-Wilk normality test was conducted for continuous variables to assess normality. If the data followed a normal distribution, the Student's t-test was applied; otherwise, the Mann-Whitney U test was used. For categorical variables, the Chi-square test or Fisher's exact test was used. To analyze the associations between PIGD score and GaitRite parameters, Spearman’s correlation test was used. ~~Baseline characteristics are presented as medians with interquartile range for continuous variables and as numbers with percentages for categorical variables.~~ To analyze the associations between potential risk factors and abnormal stereopsis in PD patients, we conducted a stepwise regression analysis (using step function in R, backward elimination and forward selection models). p-values < 0.05 were considered significant.

**Comment 18:** (Results) Table 1: The analyzed data should include the confidence interval of the mean in both groups, which is essential for the study's transparency and internal validity.

**Response 18:** We agree with the reviewer’s thoughtful comment. We added a confidence interval for the variables that followed normality and were presented as means.

**Table 1.**

| Age, y | 69.7 [68.57 – 70.83] | 70.0 [68.01 – 72.06] | 69.8 [68.80 – 70.76] | 0.779 |
| --- | --- | --- | --- | --- |
| SVLT delayed recall (z-score) | -0.6 [-0.75 – -0.43] | -0.7 [-0.99 – -0.37] | -0.6 [-0.75 – -0.47] | 0.589 |
| COWAT animal (z-score) | -0.3 [-0.41 – -0.1] | -0.3 [-0.59 – -0.07] | -0.3 [-0.41 – -0.14] | 0.645 |

**Comment 19:** (Results) Table 2: The analyzed data should include the confidence interval of the mean in both groups, which is essential for the study's transparency and internal validity. Besides, the statistical test used must be stated in the legend.

**Response 19:** Thank you for providing this insight. We have revised Table 2.

**Table 2.**

|  | PD_NL  (n = 185) | PD_AS  (n = 55) | *p-* value |
| --- | --- | --- | --- |
| Subscale of axial and gait | 5.00 [4.00, 6.00] | 6.00 [4.00, 8.00] | 0.063 |
| Subscale of resting tremor | 1.00 [0.00, 3.00] | 1.00 [0.00, 3.00] | 0.892 |
| Subscale of postural tremor | 1.00 [0.00, 2.00] | 1.00 [0.00, 2.00] | 0.311 |
| Subscale of tremor | 2.00 [1.00, 4.00] | 2.00 [0.50, 5.00] | 0.601 |
| Subscale of rigidity | 4.00 [2.00, 6.00] | 4.00 [2.00, 6.00] | 0.702 |
| Subscale of bradykinesia left | 6.00 [4.00, 8.00] | 6.00 [4.50, 7.00] | 0.804 |
| Subscale of bradykinesia right | 5.00 [3.00, 7.00] | 5.00 [4.00, 7.00] | 0.350 |
| Subscale of bradykinesia both | 10.81 [10.17 – 11.44] | 11.18 [9.92 – 12.44] | 0.380 |
| **UPDRS III sum** | 23.00 [17.00, 29.00] | 24.00 [18.00, 28.00] | 0.528 |

Values are presented as mean [Confidence interval] or median [Interquatile range]. The P-value was obtained using the Student’s t-test if the data followed a normal distribution; otherwise, the Mann-Whitney U test was used. Abbreviations: PD_NL = Parkinson’s disease with normal stereopsis; PD_AS = Parkinson’s disease with abnormal stereopsis; UPDRS = Unified Parkinson's Disease Rating Scale.

**Comment 20:** (Results) Line 186: Differences in all parameters were statistically insignificant. The same observation for the term “statistically”.

**Response 20:** We have removed the term “statistically” to reflect the reviewer’s comment.

**Results**

Supplementary table 1 shows the analysis of gait parameters between the two groups. Mean velocity was 85.41 and 83.77 centimeters per second, respectively (p = 0.602). Mean cadence was 106.71 and 107.22 steps per minute, respectively (p = 0.714). Differences in all parameters were insignificant. ~~Table 3 shows the analysis of gait parameters between the two groups. Mean velocity was 85.41 and 83.77 centimeters per second, respectively (p = 0.602). Mean cadence was 106.71 and 107.22 steps per minute, respectively (p = 0.714). Differences in all parameters were statistically insignificant.~~

**Comment 21:** (Results) Table 3: the same recommendations for table 2 and 3 regarding the 95% CI and statistical test used.

**Response 21:** We appreciate the reviewer’s insightful comments and suggestions. Although all variables, except for velocity, were continuous variables that did not follow a normal distribution, we initially presented the data as means and standard deviations to aid reader understanding. However, the p-values were calculated using the Mann-Whitney U test. In agreement with the reviewer’s suggestion, we have now revised the non-normally distributed variables to median and interquartile range, while normally distributed variable is now presented as mean and confidence interval.

**S1 Table.** **Analysis of gait parameters across groups with normal and abnormal stereopsis**

|  | PD_NL  (n = 185) | PD_AS  (n = 55) | *p-*value |
| --- | --- | --- | --- |
| Velocity (cm/s) | 85.41 [82.56 – 88.27] | 83.77 [77.53 – 90.01] | 0.602 |
| Cadence (steps/min) | 106.60 [99.30, 113.60] | 106.10 [100.20, 114.90] | 0.714 |
| Step length differential (cm) | 1.70 [0.81, 3.05] | 2.20 [1.27, 3.22] | 0.124 |
| **Left** |  |  |  |
| Step time (seconds) | 0.56 [0.53, 0.60] | 0.56 [0.51, 0.60] | 0.461 |
| Swing time (seconds) | 0.440 [0.38, 0.42] | 0.39 [0.36, 0.42] | 0.607 |
| Stance time (seconds) | 0.72 [0.68, 0.78] | 0.73 [0.67, 0.79] | 0.864 |
| Single support time (seconds) | 0.40 [0.37, 0.42] | 0.40 [0.37, 0.41] | 0.533 |
| Double support time (seconds) | 0.32 [0.28, 0.37] | 0.33 [0.27, 0.38] | 0.661 |
| Step length (cm) | 48.75 [42.24, 55.10] | 48.20 [38.75, 54.26] | 0.346 |
| Stride length (cm) | 97.86 [85.30, 110.64] | 96.50 [78.36, 106.88] | 0.292 |
| CV of step length | 6.30 [4.90, 8.10] | 6.50 [5.10, 8.10] | 0.600 |
| CV of stride length | 5.00 [4.00, 6.20] | 5.20 [4.30, 6.35] | 0.337 |
| **Right** |  |  |  |
| Step time (seconds) | 0.56 [0.53, 0.60] | 0.56 [0.53, 0.59] | 0.997 |
| Swing time (seconds) | 0.40 [0.37, 0.42] | 0.40 [0.37, 0.42] | 0.817 |
| Stence time (seconds) | 0.73 [0.68, 0.79] | 0.72 [0.66, 0.79] | 0.657 |
| Single support time (seconds) | 0.40 [0.38, 0.42] | 0.39 [0.36, 0.42] | 0.352 |
| Double support time (seconds) | 0.32 [0.29, 0.37] | 0.33 [0.28, 0.38] | 0.683 |
| Step length (cm) | 48.96 [42.82, 55.21] | 47.49 [39.23, 53.27] | 0.251 |
| Stride length (cm) | 98.57 [85.56, 111.32] | 96.46 [78.51, 107.10] | 0.333 |
| CV of step length | 6.20 [4.90, 8.20] | 6.50 [5.05, 8.80] | 0.350 |
| CV of stride length | 5.00 [3.90, 6.40] | 5.00 [4.15, 6.90] | 0.307 |

Values are presented as mean [Confidence interval] or median [Interquatile range]. The P-value was obtained using the Student’s t-test if the data followed a normal distribution; otherwise, the Mann-Whitney U test was used. Abbreviations: PD_NL = Parkinson’s disease with normal stereopsis; PD_AS = Parkinson’s disease with abnormal stereopsis; CV = Coefficient of Variation. Values are presented as mean ± standard deviation.

**Comment 22:** (Results) Lines 191-5: In the text (Table 4 shows the risk factor of abnormal stereopsis in PD. In the association analysis, a high PIGD subtype score (odds ratio 2.65, p-value 0.004) was associated with abnormal stereopsis after adjusting for UPDRS I score and abnormal SVLT result.), it is unclear which measure is significantly associated with stereopsis – was the binary definition of PIGD subtype (yes or not) or the numeric value of the PIGD score?

**Response 22:** Thank you for your valuable feedback. The numeric value of the PIGD score is significantly associated with abnormal stereopsis. Since the subtyping itself is calculated based on the ratio of the mean Tremor score to the mean PIGD score, it is reasonable to assume that a higher PIGD score increases the likelihood of being classified as the PIGD subtype. Therefore, in some parts of the discussion, we may have used PIGD score and subtype interchangeably.

**Comment 23:** (Results) The authors performed a logistic regression with stepwise modeling. What covariates were risk factors at the end? Why at the final exposition the authors state that PIGD score was adjusted for the UPDRS I score and abnormal SVLT result? Ideally, all the odds ratios should be presented. As a suggestion, a forest plot could be included containing all the variables used in the regression analysis and respective odds ratios.

**Response 23:** We are grateful for the constructive suggestions provided by the reviewer. We have reviewed the reviewer’s comments and removed variables that were not validated from the initial set (before revision) of variables (Age, Sex, disease duration (months), Education (years), hypertension, diabetes, History of visual hallucination, History of orthostatic dizziness, History of urinary incontinence, History of constipation, History of falling, History of freezing of gait, History of anosmia, Hoehn and Yahr stage, UPDRS part I, UPDRS part II, UPDRS part III, Tremor score, PIGD score, Abnormal result of Digit span backward, Mini Mental State Examination, Rey CFT copy and recall, COWAT: animal, Phonemic word fluency, and Stroop test color reading). The revised set (after revision) of variables were: age, sex, education years, hypertension, diabetes, presence of orthostatic hypotension, Hoehn and Yahr stage (HYS), UPDRS part 1, UPDRS part 2, UPDRS part 3, abnormal Mini-Mental State Examination, abnormal digit span backward, abnormal SVLT, abnormal Rey-Osterrieth Complex Figure Test (copy, delayed recall), abnormal Controlled Oral Word Association test (animal, phonemic), abnormal Stroop Color Reading test, tremor score, PIGD score and Non-Motor Symptom Scale total score. We conducted a logistic stepwise regression using the R’s built-in function, step(). The final model included the three variables shown in Table 4, which were consistent with the initial manuscript’s model: UPDRS I, Abnormal SVLT, and PIGD score.

**Materials and methods**

To analyze the associations between potential risk factors and abnormal stereopsis in PD patients, we conducted a ~~binary logistic~~ stepwise regression ~~selection~~ analysis (using step function in R, backward elimination and forward selection models). p-values < 0.05 were considered significant. The eliminated variables were: age, sex, education years, hypertension, diabetes, presence of orthostatic hypotension, Hoehn and Yahr (HY) stage , UPDRS part II, UPDRS part III, abnormal Mini-Mental State Examination, abnormal digit span backward, abnormal Rey-Osterrieth Complex Figure Test (copy, delayed recall), abnormal Controlled Oral Word Association test (animal, phonemic), abnormal Stroop Color Reading test, tremor score, and Non-Motor Symptom Scale total score. The variables selected in the regression model were UPDRS part I, abnormal Seoul Verbal Learning test, and PIGD score.

According to our consultation with the statistician, there are methodological differences between ‘stepwise regression analysis’ and ‘univariable and multivariable regression analysis.’ Although the univariable table was not presented in manuscript, we have added it to our response for clarity and transparency.

**Comment 24:** (Results) The authors must discuss the study biases and state if there was any missing data.

**Response 24:** We agree with the reviewer’s suggestion and have made the corresponding changes in the manuscript. We have addressed the potential bias in the interpretation of Hoehn and Yahr stage or RCFT in Table 1 in the discussion. Additionally, there was no missing data in our study, because only patients who completed all tests were included.

**Materials and methods**

Outcomes, covariates, missing data

Our primary outcome was the presence of abnormal stereopsis in patients with PD. The secondary outcome was subjective autonomic symptoms, and the covariates included age, sex, hypertension, diabetes, duration of symptoms, presence of orthostatic hypotension, and results of cognitive function tests related to vision. Because only patients who completed all tests were included, there was no missing data.

**Comment 25: (**Discussion**)** Overall, the text thoroughly discusses the research findings, but it lacks flow. The authors jump from one issue to another, confusing the reader.

**Response 25:** We appreciate the reviewer’s insightful comments and suggestions. We tried to achieve a more cohesive and logical progression in the revised version.

**Comment 26:** **(**Discussion**)** Line 231: “Table 1, the Rey complex figure copy score differs between the two groups, suggesting a possible association with cognition…”. The authors should consider the nature of the Rey Complex figure: this cognitive test is highly dependent on visual skills, and the stereopsis may have biased the results. This must be discussed. Considering the short mean duration of the disease (9 months) in this sample, it is not expected to have many demented patients.

**Response 26:** We agree with the reviewer’s thoughtful comment. In line with the reviewer’s comments, we have revised it as follows.

PIGD subtype patients are at a higher risk of cognitive decline [20, 24]. Abnormal stereopsis increases the risk of dementia conversion in PD [25, 26]. As shown in Table 1, the Rey complex figure copy score differs between the two groups, suggesting a possible association with cognition, consistent with the marked cognitive decline observed in PIGD patients [27, 28]. ~~However, in logistic regression analysis, cognitive function tests did not increase the risk of stereopsis abnormality~~. However, the possibility that abnormal stereopsis may have influenced the Rey Complex Figure Copy Test should also be considered. ~~However, i~~ In logistic regression analysis, no cognitive function tests ~~did not~~ increased the risk of stereopsis abnormality. Nevertheless, the abnormal Seoul verbal learning test was selected in the model with a p-value of 0.06. Although this was not significant, it could potentially show meaningful results under different conditions, such as with a larger sample size. ~~PIGD subtype may worsen stereopsis abnormalities and then cognitive function.~~

**Comment 27:** **(**Discussion**)** Line 235: “PIGD subtype may worsen stereopsis abnormalities and then cognitive function.”- PIGD subtype is a classification for PD subtypes (highly controversial nowadays).

**Response 27:** Thank you for your valuable feedback. In response to reviewer’s comments, we have deleted the sentence.

**Comment 28: (**Discussion**)** Being a PIGD subtype patient means that he or she has a higher burden of postural instability and gait disorders compared to tremor-dominant patients. This subtype is also associated with a higher decline in cholinergic function from the Meynert nucleus and PPN, which is linked to cognitive decline and gait disorders. Therefore, it's not the PIGD subtype itself that worsens these symptoms, but rather the neurotransmitter dysfunction associated with this subtype. Stereopsis may be one of the consequences of this dysfunction, linked with dopaminergic denervation in several anatomical points. The authors should revise the statement and discuss this appropriately.

**Response 28:** We thank the reviewer for the thorough review and for raising these important points. We agree with the reviewer's opinion that both stereopsis and the PIGD subtype may be associated with cholinergic denervation, and we have revised the introduction and discussion in line with this.

**Comment 29:** **(**Discussion**)** Abnormal stereopsis may be an early symptom in PD. However, in this sample, the group with abnormal stereopsis had a higher HY score. Despite the fact that HY was not a risk factor for abnormal stereopsis, the authors should discuss it in light of the literature findings.

**Response 29:** We acknowledge the reviewer’s comments and have carefully addressed them below. It is true that the HY stage reflects the progression of Parkinson's disease, but both groups consisted of early-stage patients with a disease duration of less than 36 months, and there was no difference in UPDRS Part 3 scores. Postural instability can influence the distinction between HY stage 2 and stage 2.5 or 3, which may explain the observed differences.

**Discussion**

Although our results showed that patients with abnormal stereopsis had higher HY scores, HY was not selected as a risk factor in the regression analysis. Also, there was no significant difference in disease duration between the two groups. Median disease duration was 12 months, indicating that they can be considered early-stage patients. However, further research is needed to determine whether stereopsis worsens in advanced patients and whether it could serve as a marker of disease progression.

Thank you for your kind and helpful comments.
