# Supplementary material for: Postural instability and gait disturbance are associated with abnormal stereopsis in Parkinson’s disease
Source: PLoS One. 2025 Jan 23;20(1):e0317935. doi: 10.1371/journal.pone.0317935 (PMC11756755; doi:10.1371/journal.pone.0317935)
Supplement: Attachment — Submitted filename: Response to Reviewers 2nd.docx [file pone.0317935.s003.docx]

Emily Chenette, Ph.D.

Editor-In-Chief

*PLOS One*

January 5, 2025

Dear Chenette:

On behalf of the authors, I would like to thank you and the reviewers for the valuable and helpful comments on our submitted manuscript, “**Postural instability and gait disturbance are associated with abnormal stereopsis in Parkinson's disease**” [PONE-D-24-29202].

We have carefully reviewed the comments and have revised the manuscript accordingly. Our responses are provided in a point-by-point manner, and changes to the manuscript are shown in yellow highlight, which are also visible through the Track Changes function.

We would be happy to make further corrections if necessary and look forward to hearing from you soon.

Sincerely,

Seong-Beom Koh, MD, PhD.

Professor, Department of Neurology and Parkinson’s Disease Center

Korea University Guro Hospital

Korea University College of Medicine

148, Gurodong-ro, Guro-gu, Seoul 08308, Republic of Korea

Tel: +82-2-2626-3169, Fax: +82-2-2626-1257, Email: parkinson@korea.ac.kr

Response to Reviewer 2

Comments to the Author

After a detailed analysis of the modifications made in the manuscript and the authors' responses to the reviewers' comments, the following major criticisms and observations are noted:

Thank you for your second review of our paper. We have answered each of your points below.

**Comment 1:** Experience with Parkinson’s Disease Terminology: While the authors made an effort to address the feedback provided, their responses reveal a certain inexperience with PD-specific terminology and clinical nuances. This lack of precision could lead to misinterpretations about the patient’s profile and, consequently, about the study’s findings, compromising clarity and reliability.

**Response 1:** We apologize for any terminological mistakes or parts that may have caused confusion in the first draft. In preparing this revised version, we have made every effort to use precise and clear terminology.

**Comment 2:** Insufficient Justification for Inclusion and Exclusion Criteria: The choice to include patients who had recently initiated treatment, even within a month, requires a more robust methodological justification. The lack of in-depth discussion on this potential interference with the primary outcome limits the study’s interpretative and methodological consistency.

**Response 2:** Thank you for your thoughtful comments. The one-month limitation was established to mitigate potential confounding factors, particularly medication effects, given our study's focus on early disease states. This temporal limitation, however, prevents our findings from being representative of the entire Parkinson's disease population and, as the reviewer noted, may limit the generalizability of our results. We have addressed these concerns in the discussion section.

**Discussion**

First, its retrospective study design and its being conducted at a single tertiary hospital are limitations. Additionally, it is possible that the enrolled patients constituted a unique cohort. We specified that the tests were conducted within one month of clinical diagnosis to minimize the influence of medication and maintain uniformity in the early stages of the disease. However, this approach limits generalization to the entire PD population, and this limitation needs to be further explored through additional research in future studies.

**Comment 3:** Weak Argument for Relying Solely on Clinical Assessment via UPDRS: While the authors rely on clinical assessment via the UPDRS to support their conclusions despite the lack of significant differences in objective gait assessment, the assertion of conducting evaluations by "experts" may not be wholly convincing, especially in light of some conceptual and terminological challenges noted in other areas of the manuscript.

**Response 3:** We thank the reviewer for the comment. Our clinical examinations were performed by an expert, and GaitRite data were presented to ensure objectivity. The clinical examination can be considered reliable as the GaitRite data showed correlation with the UPDRS PIGD sub score. While previous studies have demonstrated that gait analysis can detect Parkinson's disease in early-stage patients, clinical assessment by trained professionals cannot be fully substituted, despite significant technological advances. Furthermore, there were methodological limitations in our gait analysis. For instance, we were unable to measure upper extremity movements, which are often observable in early-stage patients. There is a possibility that stereopsis abnormalities may be more closely associated with upper body rather than other body parts.

**Discussion**

Second, additional gait analysis using GaitRite did not reveal any differences between the two groups. Recent studies have demonstrated that combining mat with wearable devices or 3D cameras enables the detection of subtle movements in early-stage patients, which can be valuable for diagnostic purposes [30, 31]. However, the equipment in our hospital, which consisted of only a mat and an analytical computer, was limited in its ability to assess certain kinetics and kinematics parameters such as upper extremity motion and center of pressure. It is possible that differences not captured by this equipment may be reflected in the UPDRS scores, which are based on human assessment. It is not impossible for spatiotemporal parameters to detect gait abnormalities in early PD; however, the parameters we measured in our gait analysis may have been insufficient to establish a connection with the risk of abnormal stereopsis. Thus, further studies utilizing gait analysis with integrated upper extremity movement assessment are necessary to validate these assumptions.

**Comment 4:** Lack of Exploration of Differences in Objective Gait Assessment and Clinical Evaluation: A significant inconsistency in the manuscript concerns the authors' contradictory arguments regarding the dissociation between the GaitRite and UPDRS III results. On the one hand, the authors argue that the GaitRite system has limitations and may not fully capture subtle aspects of gait and postural instability in PD patients, which could explain why it did not yield significant differences between groups. However, they also assert that the GaitRite results correlate with the UPDRS scores, suggesting that they capture relevant aspects of gait impairment. This contradictory stance weakens the credibility of the interpretation and leaves readers uncertain about the reliability of the GaitRite data. Another critical aspect that requires further discussion in the manuscript is the finding related to the single support measure, which is a key metric in assessing postural instability. The authors reported a correlation between single support time and the UPDRS for one leg but not for the other. This asymmetry raises important questions that the authors did not address, particularly regarding the potential role of early-stage PD asymmetry in influencing the observed results.

**Response 4:** We thank the reviewer for the meticulous review. Studies using wearable devices or 3D cameras have demonstrated the potential to capture subtle movements in early-stage patients, making them valuable for diagnosis. However, our equipment is limited to checking spatiotemporal parameters based solely on foot trajectories. It lacks the capability to assess upper limb movements, such as abnormal arm swings, which are commonly observed gait abnormalities in early-stage PD. This limitation may account for the differences between clinical examination and gait analysis results. Regarding the asymmetric differences in single support time shown in Table 3, since there was no comparison with a control group, we cannot determine whether this represents an abnormality detectable only by GaitRite and not by clinical observation, or if it is a measurement error of the GaitRite system.

**Discussion**

Second, additional gait analysis using GaitRite did not reveal any differences between the two groups. Recent studies have demonstrated that combining mat with wearable devices or 3D cameras enables the detection of subtle movements in early-stage patients, which can be valuable for diagnostic purposes [30, 31]. However, the equipment in our hospital, which consisted of only a mat and an analytical computer, was limited in its ability to assess certain kinetics and kinematics parameters such as upper extremity motion and center of pressure. It is possible that differences not captured by this equipment may be reflected in the UPDRS scores, which are based on human assessment. It is not impossible for spatiotemporal parameters to detect gait abnormalities in early PD; however, the parameters we measured in our gait analysis may have been insufficient to establish a connection with the risk of abnormal stereopsis. Thus, further studies utilizing gait analysis with integrated upper extremity movement assessment are necessary to validate these assumptions.
